# Implementing the Good Participatory Practice Guidelines in the Girls Achieve Power Trial in South Africa

**DOI:** 10.1177/2158244018809149

**Published:** 2018-10-29

**Authors:** A. Kutywayo, C. S. Yah, N. P. Naidoo, M. Malotana, S. Dyani, S. Mullick

**Affiliations:** 1 Wits Reproductive Health and HIV Institute, University of the Witwatersrand, Johannesburg, South Africa; 2 Grassroot Soccer, Cape Town, South Africa; 3 Sonke Gender Justice, Johannesburg, South Africa

**Keywords:** stakeholder engagement, adolescents, good participatory practice, ecological model

## Abstract

The Good Participatory Practice (GPP) guidelines provide a framework for stakeholder engagement within clinical trials, to ensure a study’s acceptability, feasibility, and improving the overall research quality; however, they have rarely been applied beyond this setting, and no literature exists on its application in adolescent research. A review of the 2011 GPP guidelines was undertaken to identify which 16 GPP topic areas could be applied and adapted for implementing an ecological asset building intervention, that is, the Girls Achieve Power (GAP Year) cluster randomized controlled trial for reducing school dropout and increasing reporting of gender-based violence in Gauteng and Western Cape province in South Africa. The 16 GPP topic areas were adapted and implemented to guide stakeholder engagement for GAP Year. We show the usability and adaptability of the GPP framework for guiding stakeholder engagement in non-clinical trials like GAP Year; however it requires adapting to respond to the unique needs of the beneficiaries.

## Introduction

Stakeholder involvement is a key component of ensuring a study’s acceptability, feasibility, enrolment, and outcome assessment as well as the design, implementation, and overall quality of the research (Bate et al., 2016; Modi et al., 2014; National Institute for Health Research, 2015). Through stakeholder engagement, international, regional, national, local partners and researchers look to each other for guidance throughout the research life cycle (Mack et al., 2013). Underscoring this participatory approach is a desire to conduct research that is designed, owned, and utilized by the community (Ellen, Wallace, Sawe, & Fisher, 2010).

Globally, there has been increased advocacy for the involvement and engagement of community stakeholders for clinical trials and health research (Molyneux & Bull, 2013). In response to this global dialogue, AVAC (AIDS Vaccine Advocacy Coalition) and UNAIDS (Joint United Nations Programme on HIV/AIDS) developed the Good Participatory Practice (GPP) guidelines (UNAIDS & AVAC, 2011) which seek to set global standards for stakeholder engagement in biomedical HIV prevention trials. First developed in 2007 and then updated in 2011, the GPP guidelines comprise 16 topic areas to guide stakeholder engagement through all stages of a biomedical HIV prevention trial (Table 1). Since 2011, the GPP guidelines have been applied more broadly to a range of disease and research areas, such as TB vaccines and drugs (Advancing Tuberculosis Vaccines for the World, 2017; Critical Path to TB Drug Regimens, 2012) and emerging pathogens (Hankins, 2016), where the guidelines constitute a framework for engaging stakeholders throughout the project life cycle (Musesengwa & Chimbari, 2017; Musesengwa, Chimbari, & Mukaratirwa, 2018). However; there are concerns that as requirements for user involvement in research expand, stakeholder participation could be undertaken simply as a tick box exercise, rather than an authentic participatory process (Holland, Renold, Ross, & Hillman, 2010; Kirby, 2004).

**Table 1:** The GPP Topic Areas, Adaptation for GAP Year, and Activities Implemented.

GPP topic areas	Adaptation for GAP Year	Implementation activities	Stage in research life cycle
Formative Research Activities Stakeholder Advisory Mechanisms	Formative Research Activities and Stakeholder Advisory Mechanisms^a^	Stakeholder mapping at national, provincial, and district level and analysis, focus group discussions with Grades 8 and 9 learners	Planning phase
Stakeholder Engagement Plans Stakeholder Education Plan Communications Plan	Stakeholder Engagement, Education, and Communication Plans^a^	Develop, implement, and update “School and Community Engagement Process” tool, buy-in meetings, Information Education and Communication material development and distribution, training sessions for coaches and data collectors, parent dialogues, text messaging platform for parents, selection of teacher champion	Throughout research life cycle
Issues Management Plan	Issues Management Plan	Develop, implement and update issues management plan, consortium meetings to deal with anticipated issues/issues already arisen	Throughout research life cycle
Site Selection	School Selection	Collaborate with national, provincial, and district Department of Basic Education for school selection	Planning phase
Protocol Development	Protocol and Tool Development	Protocol, Year 1 curriculum and baseline survey development, pre-/post-question development	Planning phase
Informed Consent Process	Informed Consent Process	Recruitment of learners by coach peers and parent dialogues	Intervention phase
Standard of HIV Prevention Access to HIV Care and Treatment Non-HIV Related Care	Referral to Local Services	Health facility selection, partner with local nongovernmental organizations, develop referral documents and clinic drop-in card	Intervention phase
Policies on Trial-Related Harms	Distress Protocol	Distress protocol development and training for coaches and data collection team	Intervention phase
Trial Accrual, Follow-Up, and Exit	Follow-Up	Qualitative data collection with learners, parents, coaches, and teacher champions	Intervention phase and close out
Trial Closure and Results Dissemination	Results Dissemination^b^	Dissemination meetings with key stakeholders, publications, reports, technical briefs, and conference presentations	Intervention phase, midterm and close out
Posttrial Access to Trial Products or Procedures	Post-Study Access for Control Sites	Control schools receive intervention if proven effective	Close out

*Note.* GPP = Good Participatory Practice; GAP = Girls Achieve Power.

a Unique to the GPP guidelines. The rest are standard practice for research and trials.

b Trial not closed, but results of baseline assessments disseminated to stakeholders.

Stakeholder engagement is now a global standard; however, little has been done to document the implementation of GPP as a framework (Hannah, Warren, & Bass, 2012; Mack et al., 2013). This article thus responds to the GPP call for increased attention to meaningful stakeholder engagement (Ellen et al., 2010; Lloyd, McHugh, Minton, Eke, & Wyatt, 2017; Shagi et al., 2008; White et al., 2011), highlighting the application and adaptability of the GPP framework beyond the clinical trial context, into adolescent research and programming in general.

GAP Year (Girls Achieve Power) is a cluster randomized control trial being implemented in 26 schools (13 intervention and 13 comparison) across two provinces (Gauteng and Western Cape [WC]). Led by a consortium of partners, Wits Reproductive Health and HIV Institute (Wits RHI), Grassroot Soccer, and Sonke Gender Justice were selected for their experience in research, delivering sports-based interventions for in and out of school youth and programming for adolescent boys, respectively. GAP Year seeks to test the implementation of an after-school intervention to empower adolescent girls as they progress in education by improving their overall health, safety, and well-being through an increase in their Educational, Health, Social, and Economic Assets. In addition, it seeks to shift gender attitudes and encourage positive behavior among adolescent boys. Informed by the “Whole Girl” approach (Erulkar, 2014) and the socioecological model (Centers for Disease Control and Prevention [CDC], 2018), the GAP Year intervention seeks to understand and address adolescents disproportionate vulnerability risks in relation to the four assets with a four-pronged intervention across the ecological model: a sports-based after-school intervention, parent intervention which includes dialogues and text messaging, linkage to care and school safety. The primary outcomes are to reduce school dropout by 20% among adolescent girls between Grades 8 and 10 and increase reporting of gender-based violence (GBV) among adolescent girls as they progress from Grade 8 to Grade 10 by 50%. Targeting Grade 8 learners with a follow-up period of 2 years, the after-school intervention is delivered by coaches between the ages of 18 and 25 years. Outcomes will be compared with 13 control schools. Although there are guiding policies and frameworks to support the health, well-being, retention, and achievement for all children and youth (Department of Basic Education [DBE], 2017; Department of Health, South Africa, 2015; Department of Social Development, South Africa, 2015; MIET Africa, 2014), adolescents still remain at risk and are the only population with increasing mortality (UNAIDS, 2016). They are also not a homogeneous group (UNFPA, 2007) and therefore require tailored approaches to both intervention and research participation.

**Figure 1:**
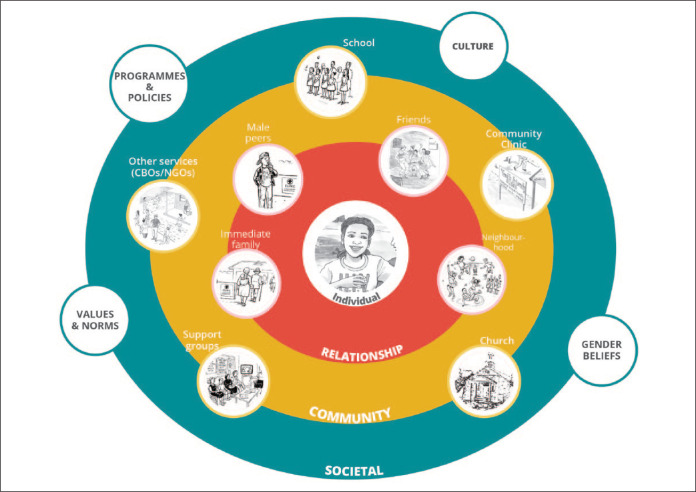
The GAP Year ecological model.

*Source*. Adapted from Centers for Disease Control and Prevention (CDC; 2018).

*Note*. GAP = Girls Achieve Power.

While adolescents are the primary recipients of the after-school intervention, it is important to form transparent and effective partnerships with parents, school staff, local health care service providers, and various Government departments (Figure 1). Engaging and informing stakeholders across all levels of the ecological model is critical for the adolescent intervention to be most effective because it ensures there is buy-in at all levels, that relevant structures are informed, reinforces messaging, and helps explore considerations for scalability.

The aim of this article is to critique how the GPP guidelines were adapted and implemented in a non-clinical trial and identify how the guidelines can be tailored to the unique needs for conducting research with adolescents.

## Method

Adopting a case study approach, we reviewed the 2011 GPP guidelines to identify and summarize which of the 16 GPP topic areas could be applied and adapted, where required, within the GAP Year randomized control trial targeting adolescents between the ages of 11 and 17 years. GAP Year study activities that could be implemented were identified and allocated to the relevant topic area to ensure that the trial adopts GPPs throughout the research life cycle. The mid-term review of GAP Year documents, site-specific work plans, and reports and reflections from site-level staff provided the results. Some gaps in the usability of the GPP guidelines were noted.

## Results

This section presents the results according to the adapted GPP topic areas for GAP Year, providing an outline of the activities implemented to date. Table 1 shows how the 16 GPP topic areas were adapted and implemented to GAP Year’s research engagement methodology.

### Formative Research Activities and Stakeholder Advisory Mechanisms

**Figure 2:**
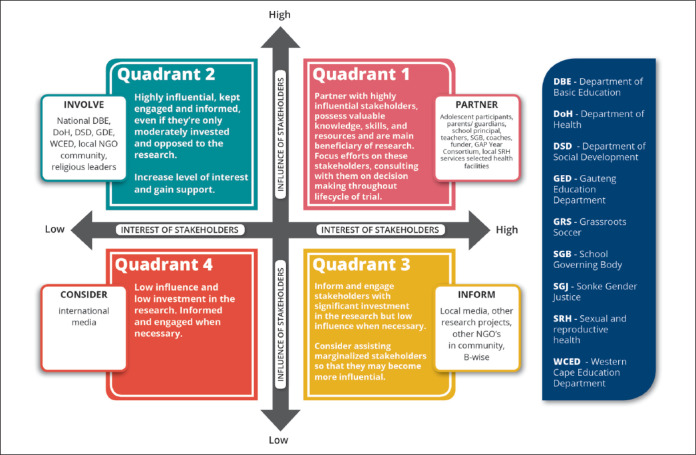
GAP Year stakeholder matrix.

*Source*. Adapted from the GPP Blueprint for Stakeholder Engagement (AVAC, 2014).

*Note*. GAP = Girls Achieve Power; GPP = Good Participatory Practice.

GPP guidelines note that formative research activities usually constitute the initial phase of stakeholder outreach and engagement to understand the local population and context (UNAIDS & AVAC, 2011). GAP Year’s first activity sought to map stakeholders, defined as “individuals, groups, organizations, government bodies, or any other individuals who can influence or are affected by the conduct or outcome of the research” (UNAIDS & AVAC, 2011). Guided by Section 2.4 of the GPP Blueprint (AVAC, 2014), stakeholders were systematically identified and ranked in terms of their influence and interest in GAP Year (Figure 2).

Referring to Figure 2, “Partner” stakeholders are vital to the success of GAP Year and should be engaged regularly and at a high level. Stakeholders to “Involve” are those who are highly influential but are only moderately interested in the research. Those in the “Inform” box have a lower influence on the success of the research and will be engaged less regularly. This process of mapping helped to determine an appropriate level of engagement for each stakeholder and where to prioritize efforts while also supporting the team to think about how a stakeholder’s interest and influence in GAP Year could be improved, moving them into the quadrant up.

Following the stakeholder mapping, GAP Year employed a variety of formal and informal stakeholder advisory mechanisms to “facilitate meaningful dialogue among research teams and stakeholders about the research” (UNAIDS & AVAC, 2011). The use of different mechanisms recognizes that needs and preferences of stakeholders vary. A stakeholder directory was developed to document their name, contact details, relation, and influence on GAP Year for future engagements.

Formal, face to face, meetings were initiated to engage the National DBE and subsequent meetings with both Gauteng and WC Provincial Education Departments. The purpose of these meetings was to present the GAP Year program, request research permission and access to the schools, and consult on school selection. Their main questions were about the content of the curriculum sessions and how it complemented the Life Orientation curriculum. Meetings followed with local district, municipalities, and school-level stakeholders discussed in the “Stakeholder Engagement” section.

In addition, 11 focus group discussions (FGD) were conducted with adolescents between the ages of 13 and 18, in Soweto (*n* = 8) and Khayelitsha (*n* = 3) townships. The FGD’s with adolescents and the coaches sought to get an in-depth understanding of the issues and challenges adolescents face in their respective communities. The FGD’s were informed by the following key areas: being an adolescent, school and community safety, important assets (health, social, education, and economic), curriculum (key topics they believe should be covered in the curriculum), and promotional incentives (design of the proposed kits for the participants [t-shirts, bags, hats, etc.] and key messages).

### Stakeholder Engagement, Education, and Communication Plans

GPP guidelines recommend developing “strategies and mechanisms for building relationships and constructively engaging with a broad range of stakeholders” to “provide relevant education to enhance research literacy” (UNAIDS & AVAC, 2011).

*Engagement*. The GAP Year consortium discussed the education, engagement, and communication needs of different stakeholders at the beginning and update regularly. Informed by the GPP Guidelines (UNAIDS & AVAC, 2011), a “School and Community Engagement Process” tool was developed with the purpose of documenting and guiding the systematic steps to be taken in engaging learners, educators, parents, local facilities, and Government stakeholders at national, provincial, and district level, recognizing their key, albeit differing, roles in the success of GAP Year. All staff were trained on this tool to ensure stakeholder engagement is standardized and systematic. One of the initial activities in the “School and Community Engagement Process” was to engage provincial government stakeholders, local health facility staff, other implementing nongovernmental organizations (NGO) as well as staff at selected schools. In response, the GAP Year consortium hosted this multisectoral meeting at one site, which provided a platform to present GAP Year, discuss areas of overlap and synergy while opening a dialogue between the research team and these key stakeholders. It is important to note that engaging stakeholders is not a one-way process; attending other NGO’s stakeholder meetings shows our support for their work and it builds trust and stronger partnerships.

Staff from selected schools were poorly represented at GAP Year’s initial stakeholder meeting, so a follow-up visit was held with schools directly. School staff who did attend emphasized that the lack of household income to buy sanitary towels was one of the many reasons for absenteeism among the female learners in their schools and perhaps GAP Year should therefore consider the provision of sanitary towels to the female participants. They also mentioned that there was an increase in female learner participation in gangs which they attributed to higher pregnancy rates among learners. The GAP Year consortium considered these issues in the planning of activities and explored the baseline data for findings to substantiate these claims. It is important to note that although national DBE have approved the research, South Africa functions as a decentralized state, that is, there is transfer of power to subnational levels, that is, provinces, districts, and schools (McIntyre, Muirhead, & Gilson, 2002). Therefore, school principals are the key decision makers regarding buy-in and access to the school environment. Consequently, individual visits to the principals or deputy principals (where principals were absent) of selected schools were critical to educate and communicate effectively about GAP Year and provide opportunities to ask questions. Most principals of intervention schools were supportive of GAP Year while others were curious about how their school was selected and anxious about the findings of the research. Each school was provided with a GAP Year information pack consisting of the GAP Year overview, baseline survey questions, and the research approval letter from Provincial DBE. Following permission from the principal for their school to be part of GAP Year, a teacher, usually the Life Orientation teacher, was selected as the school’s GAP Year teacher champion. As the pivotal person between the GAP Year team and the school staff, the teacher champion guided GAP Year implementation activities in their school which included supporting learner recruitment, arranging room availability, and supporting learner retention by being a GAP Year advocate.

*Education*. As part of stakeholder education and to build the research capacity of the coaches and the data collection team, training sessions were held regularly, equipping them with the basics of a randomized control trial, ethics training, and understanding concepts such as informed consent, randomization, contamination, and control/intervention groups. This training equipped coaches to address questions and challenges that arose during learner recruitment and data collection related to the importance of research procedures such as the wording used when recruiting the control schools. This was a critical aspect to ensure all GAP Year staff understood these processes.

GAP Year’s primary focus is on adolescents between the ages of 11 and 17 years, which has inherent challenges around informed consent. Being minors, ethics review committees have stringent guidelines around engaging adolescents in research studies. Therefore, the need for parental involvement and education is critical throughout the lifecycle, especially at the beginning when parental consent was required for adolescent participation in GAP Year. Subsequently, the team hosted two parent dialogues per school, that is, before and after the first year of intervention. There were joint parent dialogues at the end of the intervention with all the parents from the intervention schools. The direct engagement between the research team and the parents provided (a) valuable opportunities to present GAP Year, (b) information regarding topics covered in the after-school program curriculum, and (c) a space to ask questions that pertained to the study implementation. Dialogues were also opportunities to garner feedback from parents on the acceptability and myths associated with the launch of new policies and programs such as the DBE policy on HIV, sexually transmitted infection (STI), and tuberculosis (TB; DBE, 2017). Parents commonly asked why GAP Year was only for 2 years and not an ongoing program for their adolescents which provided us with more opportunities to enhance their research literacy and explain GAP Year was a research trial. Parents commented that they liked to be kept involved in GAP Year to know what their adolescents were learning. In some dialogues, a coach led parents through a practical after-school practice to give them an idea of what their adolescents would experience during the after-school intervention.

Information leaflets were developed for parents, schools, and broader stakeholders that provided an overview of GAP Year and outlined their role as parents and schools in the research. Available in Xhosa and English, these information materials were distributed to educate the aforementioned about GAP Year.

*Communication*. Schools and families usually share the responsibility of providing sexuality education to adolescents but many parents lack the skills, knowledge, and confidence to relay messages and therefore need information, motivation, and strategies to help them reinforce the appropriate messages (Pop & Rusu, 2015). With this in mind, and to facilitate ongoing communication, a text messaging platform was set up where parents with a mobile telephone received a bi-weekly push message, outlining the topics their adolescent child would be exposed to during the GAP Year after-school intervention for each week. This provided parents with regular information on what their adolescents were learning, ideally leading to a conversation at home between the adolescent and their parent(s). Parent dialogues also helped the communication flow between parents and the GAP Year team.

### Issues Management Plan

Planning for unexpected developments that may emerge before, during, or after the research is recommended by the GPP guidelines (UNAIDS & AVAC, 2011). The GAP Year team proactively identified potential issues that could impact the research. For example, the challenge of recruiting in the control schools, selection bias, the safety challenges of a township environment, and the challenges of informed consent from minors.

Challenges were anticipated for the recruitment in control schools as there would be no direct benefit to the participants because they would not receive the after-school intervention. To enable correct and clear messaging, a recruitment brief was drafted, highlighting the long-term benefits of GAP Year’s research, noting that if the research proved to show an effect of the after-school curriculum on adolescents progressing through school, the control schools would be the first to receive the intervention. The issue of selection bias was mitigated, albeit not completely, by documenting the reasons for refusal to participate to better understand the profiles of those who did not participate. GAP Year’s research is taking place in a township environment that poses safety and security issues to the learner participants as well as the GAP Year team. This was addressed by working with implementing partners who are familiar with these locations, as most of the staff come from these communities. To mitigate the challenges of informed consent for minors, parent dialogues were planned and the text messaging platform was used to inform parents about each stage of the GAP Year program, from recruitment to graduation. This was in an effort to improve transparency and communication with parents, thereby improving recruitment and retention.

During implementation, an additional challenge arose around the safety of learners getting home safely after the after-school intervention. Following discussions with the consortium and site-level staff, a transport company was screened and briefed to provide safe, reliable transport for learners and coaches to their homes, following the after-school intervention.

### School Selection

School selection is the process by which high-level stakeholders such as the national, provincial, and district DBE and GAP Year consortium collaborate to evaluate the schools readiness for the intervention (UNAIDS & AVAC, 2011). The GAP Year team employed a multistep process to determine school selection. First, a set of predefined inclusion and exclusion criteria was developed to select and randomize 26 schools. The inclusion criteria was as follows: public high schools in Tembisa, Soweto, and Khayelitsha; mixed sex; not had exposure to any assets building intervention in the past 6 months; and quintiles 1 to 3. The exclusion criteria was as follows: public high schools, catering for learners with special needs or learning difficulties, private high schools, single-sex high schools, high schools that have been exposed to similar interventions within the last 6 months, and quintile 4 to 5 public high schools.

Second, consultations were conducted with the Education District Life Skills and HIV/AIDS Directorates, and schools were then selected based on the inclusion and exclusion criteria. Additional engagements were undertaken with various national and provincial directorates (Education, Health Promotion, Coordinator of HIV and Life Skills Programs, Social Cohesion and Equity in Education, School Safety) and research officials to enhance the acceptability and adoption of the research and get their buy-in. The stakeholders reflected on the research outcomes before approving the townships.

Third, the GAP Year team was introduced to the selected school principals by the Education District Directorate officials where further consultations took place. The adoption of the study in each school was based on the prerogative of the principal and the School Management team. Letters of study acceptance were obtained from the adopting principals, as well as the selection of a school champion.

### Protocol and Tool Development

GPP guidelines note that stakeholders can provide meaningful input into the protocol and other tools related to the research (UNAIDS & AVAC, 2011).

*Protocol*. The overall design of GAP Year was conceptualized by all consortium partners; however, being a research trial, Wits RHI took the lead on the development of the protocol. The protocol design took an interactive multistep process. An ecological, learner-centered study design and intervention approach (Figure 1) was adopted for GAP Year, which guided the development of the study protocol. The protocol included the selection of the learners’ grade, recruitment and informed consenting processes, study tool development, study design, outcome measures, timing and duration of the after-school intervention, and adapting of the language to ensure it is culturally appropriate for the intervention.

*Year 1 and 2 curriculum*. The Year 1 and Year 2 after-school curricula were also collaboratively developed by the GAP Year consortium. Both GAP Year curricula are aligned to the South African Curriculum Assessment Policy Statements (CAPS; DBE, 2018) and use the platform of soccer to address the barriers adolescents face related to HIV prevention, gender equality, safety, life skills, and access to health services. Prior to the development of the curricula, learners and coaches (18-25 years) were involved in formative research which assessed key issues around what it means to be an adolescent, what asset is most important to them, participation in sexual and reproductive health interventions, and identifying any perceived gaps in these interventions. This formative research was used to inform the type of content to include in Year 1 and Year 2.

Year 1 curriculum development started by identifying themes and topics to be included in the curriculum and assigning assets to the themes to ensure that the four assets (educational, health, social, and economic) were well covered. Year 1 is a single-sex after-school curriculum that comprises 22 sessions on HIV, sexually transmitted diseases, sexual reproductive health and rights, understanding violence, sex and gender, relationship-power and leadership, decision making, personal development, budgeting, and developing of business plans as well as the session to get to know your local-service providers. Some content and activities were drawn from *Skillz Street* (Grassroot Soccer, 2013), *Youth Changing the Rivers Flow* (SAfAIDS and Sonke Gender Justice, 2016), and *One Youth Can* (Sonke Gender Justice, 2016) curricula. Each existing curricula underwent rigorous scrutiny and review to extract and examine the components that fit into the overall objectives of the GAP Year study. GAP Year–specific outcomes were then linked to each of the 22 sessions as per the research aims.

The Year 2 mixed-sex curriculum builds on the Year 1 curriculum, “Generation Skillz” (Grassroot Soccer South Africa, 2010), the DBE’s CAPS (DBE, 2018), and addresses the key issues arising from GAP Year’s baseline data. It was developed using the intervention mapping approach (Bartholomew et al., 2016), which used theory and evidence as foundations for taking an ecological approach to assessing and intervening in health problems. Intervention mapping takes a series of six iterative steps: develop a logic model of the problem, identify GAP Year outcomes and objectives, review theories and practical strategies with evidence of behavior change, curriculum production drawing from the consortium’s existing curricula, develop curriculum training and implementation plan, and finally, evaluate the final product. The Year 2 curriculum also takes into account adolescent neurobiology exploring how adolescents respond to content and learning styles and taking into account the plasticity of the brain as they are developing.

The two curricula are structured to achieve specific outcomes at individual (boys/girls), schools, and community levels specifically the reduction in school dropout, reporting of sexual and gender-based violence, and uptake of health care services. Each asset in the curricula are logically linked and seeks to achieve the primary objectives that are aimed at reducing school dropout rates, increasing reporting of GBV, uptake of services, and promoting health well-being.

The coaches provided invaluable insights into the design and pilot of the curricula, suggesting images to be used, terminology to be adapted, and additional topics, like mental health, to be included. They provided valuable input on the structure of the curriculum and the wording of certain questions, for example, use of the word “contraception” rather than “family planning” and “practices” rather than “lessons,” ensuring it was age and context appropriate. Coaches helped assess whether the activities would be feasible in their context and the curriculum developer made amendments accordingly. This was a critical process of stakeholder engagement, as it is especially important when working with adolescents that terminology and design of materials are age and context appropriate as well as appealing.

### Informed Consent Process

The informed consent process is relevant to GPP because stakeholders can help develop locally acceptable and effective informed consent procedures and materials (UNAIDS & AVAC, 2011).

Once schools were selected, peer coaches from the same community led the recruitment of learners for each school. With the permission of the principal, teacher champion, and classroom teacher, learners were approached in their classrooms and given a brief overview of GAP Year, using a recruitment brief, to ensure clear and consistent messaging across schools and coaches. All learners who were interested in participating were provided with a consent form, available in English or Xhosa. The consent form was explained to the learners during recruitment, reinforcing the need for their parents to also sign the form and consent to their participation in GAP Year. Providing potential participants with enough information about the intervention is key for them to make an informed decision about participation. Questions commonly raised by the adolescents were about transport home, the cost of participation in the program, the provision of food, and whether the activities were very physical. In the days following, the coaches returned to the school to collect completed consent forms and inform the learners, now GAP Year participants, when the after-school intervention will start.

### Referral to Local Services

The GPP guidelines note that participants should have access to local health care services and the research teams should facilitate optimal referrals and linkages to care (UNAIDS & AVAC, 2011). GAP Year aims to increase adolescent’s access to sexual and reproductive health and post-violence care services, recognizing their health needs. In line with GPP guidelines, GAP Year has established linkage mechanisms with selected health facilities to create a smooth referral pathway for adolescents into HIV and non-HIV related care. This mechanism includes the measurement of linkage to care and service uptake by adolescent participants. Following the engagement and partnership with six facility managers at local health facilities in WC, GAP Year drop-in boxes have been installed and participants are encouraged to anonymously document what service(s) they received at the facility and rate the facility using the drop-in card. A GAP Year Map has been developed to assist adolescent participants to locate their nearest clinics, hospitals and places of safety (police stations) and highlights the six clinics that have the GAP Year drop-in boxes. The map provides the reader with National Support service contact details for the following issues: depression, alcohol and drug abuse, sexual, and emotional or physical abuse. During the after-school intervention, the GAP Year Map, B-Wise^1^, and the She Conquers roadmap to services^2^ have been actively promoted to encourage health-seeking behavior and uptake of local services. These online, adolescent-friendly platforms provide accurate health information at their fingertips. In addition, GAP Year have partnered with Médecins Sans Frontières in WC, to conduct the “Know your service provider” session during the after-school intervention. The team have also partnered with TB/HIV Care to conduct HIV testing services at the GAP Year graduation events, recognizing the barriers adolescents face in accessing facility services.

### Distress Protocol

In the original GPP guidelines, this topic area was “Policies on trial related harms” which has been adapted to “Distress Protocol” for the GAP Year research. To complement the referral processes, a distress protocol was adapted from Draucker, Martsolf, and Poole (2009), to guide the site-level staff on how to respond if a case of violence, abuse, or emotional distress is reported. As per requirements of a trial, a distress protocol has to be developed to address the issue of a social harm, which is an adverse social consequence as a result of participating in a trial. The distress protocol was developed using content from a social worker and provides a list of services that the participant may be referred to. All site-level staff have received training on this tool.

### Results Dissemination

Disseminating results from the research is a transparent process, essential for building trust and laying positive foundations with stakeholders (UNAIDS & AVAC, 2011). In line with this, GAP Year results dissemination will be a continuous process through workshops, and presentation meetings with stakeholders in Quadrants 1 and 2 of Figure 2. These engagements will not all take the same format or share the same depth of information due to each stakeholder’s different needs.

Thus far, mid-term dissemination meetings have been held with provincial, district, and school stakeholders in the WC. This involved a recap of the GAP Year trial outcomes and intervention approach, progress to date, and the findings of baseline surveys conducted with learners. An overview of the Year 2 curriculum was shared as well as responding to questions around the data findings. The aim of this meeting was to ensuring transparency, accountability, and buy-in for Phase 2 as well as facilitating changes to their programming and policy. To respect the intervention and control schools, an agreement has been made to share baseline and end line findings with the school principals prior to publishing the data. The GAP Year team will also formally disseminate results to the scientific community through publications, reports, technical briefs, and conference presentations.

## Discussion

We found that the GPP guidelines provide a useful framework to guide stakeholder engagement in GAP Year’s research among adolescents in South Africa.

Our review of the GPP framework identified that most of the GPP topic areas were applicable to GAP Year; however, adaptations were required for the study design, location, tools, and target audience. We tailored them specifically to meet the unique needs of South African adolescent participants in the following ways. We planned and executed engagements with stakeholders at all levels of the ecological model recognizing that most stakeholders impact the research outcomes for adolescents. FGDs with adolescents and workshops with coaches took place to discuss tool development, messaging, and information education and communication material content. The recruitment of adolescents was conducted by peer coaches (peer to peer), and a mobile platform for adolescents to get accurate health information (B-Wise) was promoted for adolescents to use. We ensured that consent forms were translated so participants could understand in their own language. A text message platform for parents was developed to share information easily and quickly about GAP Year and keep them involved.

We found that systematically planning and implementing stakeholder engagements using the GPP framework helped elicit feedback and requests for further information from various stakeholders. We reflect on the impact of using the GPP framework on the activities implemented. Referring to stakeholder engagement, education, and communication, we found that selecting a GAP Year teacher champion per school facilitated access to the school, as they acted as advocates for GAP Year, often promoting GAP Year to other stakeholders. In addition, where the teacher champion was active and involved, the retention and participation of learners was greater. Hosting a large multisectoral stakeholder meeting was appropriate for presenting GAP Year to a bigger group of people as it allowed questions to be asked and helped identify areas of overlap and synergy. For example, during one initial meeting, a few local facilities and NGOs noted that they already work in some of the selected GAP Year schools which may contaminate the research. Knowing this early on was helpful, and subsequently, we have aligned school engagement plans and layer with these stakeholders. We have partnered with two of these NGOs to provide GAP Year participants with HIV testing services and a “Know your service provider” session.

We recognize that there is still progress to be made on the continuum toward true partnership with adolescents, the local community, and other key stakeholders as the process is ever evolving. The use of GPP as a framework not only helped the research outcomes but also built broad-level support for possible future nonclinical research with adolescents.

We have identified that we need to develop strategies to communicate with parents who do not have access to a mobile phone and ensure meaningful involvement with religious leaders in GAP Year’s research. Referring to issues management, being prepared for anticipated issues through discussions with the consortium and site-level staff, allowed the team to implement planned strategies effectively when the issues emerged.

While most of the nine topic areas adapted for GAP Year are standard practice for trials and research, we found that five topic areas are unique to the GPP guidelines (UNAIDS & AVAC, 2011): (a) Formative Research Activities, (b) Stakeholder Advisory Mechanisms, (c) Stakeholder Engagement, (d) Stakeholder Education, and (e) Stakeholder Communication Plans. These five elements of the GPP guidelines, in particular, have improved the implementation, retention, and impact of GAP Year’s research to date by supporting the team to think systematically about stakeholder engagement. Providing a clear structure from the onset, these two topic areas particularly assisted the team in planning and engaging stakeholders from all levels of the ecological model, especially adolescents, tailoring our approaches to meet their needs.

We reinforce that stakeholder engagement should be an active process throughout the life cycle of any research, trial, or program, allowing for corrective action and adjustments to be made, keeping up to date with the needs of the key stakeholders in real time. This is particularly important when working with adolescents where their needs and opinions may change more rapidly.

We conclude that asking questions and responding to information needs is a meaningful way of participatory practice, one that isn’t a tick box exercise, and serves to empower communities and participants. At the time of writing, this article is one of the first papers documenting the adaptation and implementation of the GPP guidelines in the context of a nonclinical trial (Musesengwa et al., 2018).

### Limitations and Future Needs

While GAP Year is seeking to engage stakeholders effectively and meaningfully, we have identified the following limitations and gaps as well as future recommendations. The components of this article rely on insider perspectives where it may be difficult to be objective. To support their reflections, it would be useful, in future, to also include the perspectives of external stakeholders. While there are monitoring and evaluation tools developed to capture, collate, and analyze stakeholder engagement data at the site level (Engagement for Impact, 2018), thus far, no validation indicators have been used to measure GAP Years implementation of the GPP guidelines. In future, we suggest that the GPP guidelines are updated to include timelines for the topic areas, indicating which take place in the planning phase, implementation phase, or close out phase or throughout the whole research life cycle. This may also support the process of constant evaluation and reevaluation of activities.

Overall, the GPP guidelines provide a clear and useful framework for implementing stakeholder engagement for any trial, research, or program. Using the adapted GPP guidelines to structure stakeholder engagement has improved the implementation, retention, and impact of GAP Year’s research to date.

## Notes

1 B-Wise is the National Department of Health’s mobi site for adolescents and young people in South Africa to primarily get health information and connect to health services in their community (B-Wise, 2018).

2 The roadmaps help the reader find the information they need around five topics—sex, pregnancy, school, future career, and safety—and refer the reader to services to support their choices. View the roadmaps here: http://sheconquerssa.co.za/roadmaps/

## Authors’ Note

The views expressed in this article are the authors own and not an official position of the institution or funder.
